# Preoperative Serum IL6, IL8, and TNF-*α* May Predict the Recurrence of Hepatocellular Cancer

**DOI:** 10.1155/2019/6160783

**Published:** 2019-10-20

**Authors:** Huayong Cai, Yu Zhang, Fanyu Meng, Chao Cui, Hao Li, Minghao Sui, Haoyun Zhang, Shichun Lu

**Affiliations:** ^1^Nankai University School of Medicine, Tianjin, China; ^2^Department of Hepatobiliary Surgery, The First Medical Center of Chinese PLA General Hospital, Beijing, China; ^3^Department of Radiology, The Second Hospital, Dalian Medical University, Dalian, China

## Abstract

**Purpose:**

As we all know, curative resection remains the only effective treatment for hepatocellular cancer (HCC). However, systemic inflammatory response syndrome always correlates with surgery, which may impose an impact on the clinical outcome of HCC patients who had undergone curative treatment. The present study is aimed at exploring the correlation between perioperative inflammatory mediators and recurrence risk of HCC.

**Methods:**

This study retrospectively included 157 histologically confirmed single HCC patients (88 patients developed HCC again) who had received radical hepatectomy between January 2016 and May 2018 at the Department of Hepatobiliary Surgery, the People's Liberation Army General Hospital (PLAGH), China. The cut-off values for predicting recurrence were determined by receiver operating characteristic (ROC) curve analysis with estimation of the Youden index. Recurrence-free survival (RFS) was assessed using the Kaplan-Meier method, and the difference was compared between groups by the log-rank test. Univariate/multivariate analysis was performed to identify independent risk factors of postoperative tumor recurrence.

**Results:**

The perioperative serum IL1, IL2, and IL10 levels showed no difference between groups, whereas the serum IL6, IL8, and TNF-*α* levels showed significant differences between groups. High preoperative serum IL6, IL8, and TNF-*α* levels were significantly associated with shorter RFS. Multivariate analysis revealed that preoperative serum IL6 > 8.45 pg/ml, preoperative serum IL8 > 68 pg/ml, preoperative serum TNF − *α* > 14.9 pg/ml, microvascular invasion (MVI), and maximum tumor size > 6 cm were independent predictors of RFS.

**Conclusions:**

The present study confirmed that high preoperative serum IL6, IL8, and TNF-*α* levels were distinctly correlated with the postoperative tumor recurrence risk of HCC patients.

## 1. Background

Liver cancer is the sixth most common cancer and the fourth leading cause of cancer death worldwide, and hepatocellular cancer (HCC) accounts for 75-82% of the cases [1]. In the past decade, though some advances had been made in the treatment of HCC, radical hepatectomy remains the only effective therapy for HCC patients, and these patients had poor outcomes. Postoperative tumor recurrence risk is the main concern of these patients who received radical hepatectomy. However, there are few significant predictors of postoperative tumor recurrence risk for HCC patients, and clinical parameters independently related to tumor recurrence risk are urgently required to improve HCC patients' outcome.

The chronic inflammatory response may play a crucial part in the evolvement of cancer, and inflammatory mediators are prominent players in these physiological processes [2, 3]. Previous studies showed that cytokines play crucial roles in the tumorigenesis of breast cancer [4, 5], lung cancer [6], gastric cancer [7, 8], and ovarian cancer [9]. Among these cytokines, interleukin-6 (IL-6) has been well characterized and is considered as an important element of the systemic immunity [10]. Some clinical studies had confirmed that preoperative serum IL-6 could predict the clinical outcome of several kinds of tumors [11–17]. In addition to IL6, interleukin-8 (IL8) and tumor necrosis factor-*α* (TNF-*α*) also played some important roles during the evolvement of cancers [18, 19]. Moreover, Porcelli et al. suggested that the serum IL8 level was significantly correlated with the responsiveness to gemcitabine/nabpaclitaxel in pancreatic ductal adenocarcinoma patients and could serve as prognostic biomarkers for those patients [20]. Furthermore, in a recent review by Yu et al., nonresolving inflammation may contribute to the development of HCC by activating invasion and metastasis, inducing genome instability and angiogenesis, promoting proliferative and survival signaling, and evading immune surveillance [21]. Based on the above findings, the serum inflammatory markers may be related to tumor recurrence, and targeting cytokines related signaling may have some potential values in the treatment of cancers mentioned above. These findings stimulated us to examine the relationship between inflammatory mediators and the clinical outcome of HCC patients.

In the present study, we aim to explore the dynamic changes of perioperative inflammatory mediators and define the relationship between inflammatory markers and the prognosis of HCC patients.

## 2. Methods

### 2.1. Patients

We retrospectively included 157 histologically confirmed single HCC patients (88 patients developed HCC again) who had received radical hepatectomy between January 2016 and May 2018 at the Department of Hepatobiliary Surgery, PLAGH, China, and this study was authorized by the PLAGH medical ethics committee.

The entry criteria are as follows: age 18 years or older, histologically confirmed single HCC, Child-Pugh class A or class B that can reverse to class A after conventional therapy, had accepted radical hepatectomy, and had complete data of perioperative inflammatory mediators. Exclusion criteria included other malignancies other than HCC, nonsurgical interventions, and lack of follow-up.

### 2.2. Data Collection

We collected the following preoperative data: demographics and laboratory examinations. Operative parameters included operative time, operative procedure, Pringle maneuver, and total blood loss. According to the histopathologic reports, we determined the tumor number and size, tumor differentiation, microvascular invasion (MVI), and cirrhotic change. The serum IL1, IL2, IL6, IL8, IL10, and TNF-*α* concentrations were quantified by the IMMULITE® 1000 Immunoassay System (Siemens Medical Solutions Diagnostics, Tarrytown, NY, USA) at preoperative and postoperative days 1, 3, 5, and 7. All data was retrospectively collected from original medical records at our hospital.

All patients had received radical hepatectomy by the same surgical team and received regular follow-up (the 1st, 3rd, 6th, and 12th months after surgery during the 1st year and every 6 months beginning in the 2nd year) in the outpatient department by the surgeon. During every follow-up, all patients received laboratory examination (such as serum alpha fetoprotein (AFP), alanine transaminase (ALT), and aspartase transaminase (AST)) and contrast-enhanced MRI examination. Follow-up data were obtained from our prospective database and through direct contact with patients and their families, and the follow-up deadline was December 2018.

### 2.3. Outcome

The present study is aimed at investigating the correlation between preoperative inflammatory mediators and the postoperative tumor recurrence risk of HCC.

The secondary objective includes investigating the change of perioperative inflammatory mediators' level and identifying the independent predictors of recurrence-free survival (RFS, the date of radical hepatectomy to the date of postoperative tumor recurrence (without regard to the recurrence pattern) or deadline (December 2018) was calculated as RFS).

### 2.4. Statistics

Continuous variables were described as the “mean ± standard deviation (SD)” or median (interquartile range), appropriately, and were compared using the Mann–Whitney *U* test or Student's *t*-test. Categorical data were summarized by frequency and were compared using the *χ*^2^ test or Fisher's exact test. The correlations between preoperative inflammatory mediators and maximum tumor size were analyzed by Pearson *r*. The RFS curves were plotted using the Kaplan-Meier method and compared by the log-rank test. The risk factors of postoperative tumor recurrence were determined by the univariate/multivariate Cox proportional hazard model. The optimal cut-off values for predicting recurrence were determined by ROC curve analysis with estimation of the Youden index. Significance was represented by *p* < 0.05, two sides. All statistical tests were done by SPSS 22.0 software.

## 3. Results

### 3.1. Baseline Characteristics

This study retrospectively included 157 histologically confirmed single HCC patients who received curative hepatectomy from January 2016 to May 2018. During the follow-up, 88 patients developed HCC again. The median follow-up period for the recurrence group or nonrecurrence group was 24.0 (16.0 to 30.0) and 24.0 (17.0 to 27.0) months, respectively.

Table 1 depicts the baseline characteristics of patients from the recurrence group or nonrecurrence group. The baseline characteristics (age, gender, Child-Pugh score, differentiation, cirrhosis, HBsAg status, operative time, total blood loss, AFP, ALT, AST, TBIL, and operative procedure) were balanced between the recurrence group and the nonrecurrence group, excepting that the maximum tumor size of the recurrence group was bigger than the nonrecurrence group (8.1 ± 3.9 versus 5.5 ± 3.2; *p* < 0.001), and MVI was more common in the recurrence group.

### 3.2. The Dynamic Changes of Perioperative Inflammatory Mediators

**Figure 1:**
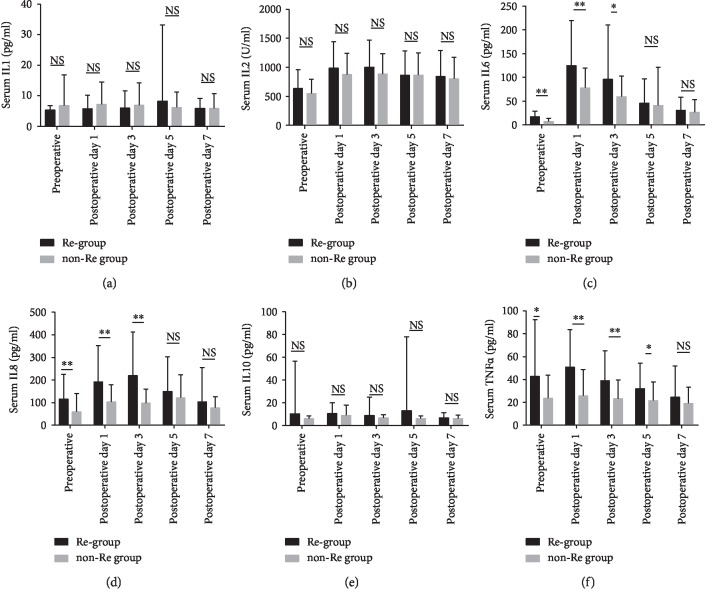
Postoperative changes in serum IL1, IL2, IL6, IL8, IL10, and TNF-*α* levels: (a) serum IL1 level, (b) serum IL2 level, (c) serum IL6 level, (d) serum IL8 level, (e) serum IL10 level, and (f) serum TNF-*α* level. ^∗^*p* < 0.05; ^∗∗^*p* < 0.001; NS: *p* > 0.05; Re: recurrence; non-Re: nonrecurrence.

The postoperative change of serum IL1, IL2, IL6, IL8, IL10, and TNF-*α* levels was shown in Figure 1. Before operation, the serum IL6, IL8, and TNF-*α* concentrations of the recurrence group were significantly higher than those of the nonrecurrence group (IL6: 16.9 ± 12.2 vs. 6.9 ± 6.8, *p* < 0.001; IL8: 115.2 ± 109.8 vs. 58.7 ± 81.2, *p* < 0.001; and TNF-*α*: 42.7 ± 49.8 vs. 23.6 ± 20.3, *p* = 0.003, respectively), but the serum IL1, IL2, and IL10 concentrations were comparable between groups (IL1: 5.3 ± 1.5 vs. 6.7 ± 10.2, *p* = 0.197; IL2: 633.2 ± 322.5 vs. 546.7 ± 247.4, *p* = 0.067; and IL10: 10.2 ± 46.2 vs. 5.7 ± 2.7, *p* = 0.806, respectively). At postoperative day 1 and day 3, the serum IL6, IL8, and TNF-*α* concentrations of the recurrence group remained higher than those of the nonrecurrence group (postoperative day 1: IL6: 124.6 ± 95.0 vs. 78.0 ± 41.2, *p* < 0.001; IL8: 191.4 ± 160.7 vs. 102.9 ± 76.1, *p* < 0.001; and TNF-*α*: 50.8 ± 32.7 vs. 25.7 ± 23.1, *p* < 0.001, respectively; postoperative day 3: IL6, 95.7 ± 114.8 vs. 6.9 ± 6.8, *p* = 0.014; IL8: 219.0 ± 193.3 vs. 97.1 ± 61.8, *p* < 0.001; and TNF-*α*: 39.0 ± 26.0 vs. 23.0 ± 16.5, *p* < 0.001, respectively), whereas the serum IL1, IL2, and IL10 levels remained similar between groups (postoperative day 1: IL1: 5.7 ± 4.5 vs. 7.1 ± 7.3, *p* = 0.14; IL2: 983.6 ± 456.3 vs. 873.6 ± 368.4, *p* = 0.105; and IL10: 10.3 ± 9.6 vs. 8.8 ± 9.1, *p* = 0.337, respectively; postoperative day 3: IL1: 5.9 ± 5.7 vs. 6.8 ± 7.3, *p* = 0.38; IL2: 1001.8 ± 469.6 vs. 883.1 ± 354.4, *p* = 0.083; and IL10: 8.5 ± 16.2 vs. 6.5 ± 2.8, *p* = 0.308, respectively). Five days after the operation, only serum TNF-*α* concentration was significantly different between groups (31.9 ± 22.4 vs. 21.7 ± 16.2, *p* = 0.002), the serum IL1, IL2, IL6, IL8, and IL10 concentrations were comparable between groups (IL1: 8.2 ± 25.0 vs. 6.2 ± 5.1, *p* = 0.502; IL2: 862.1 ± 421.7 vs. 866.4 ± 382.7, *p* = 0.948; IL6: 45.6 ± 51.2 vs. 41.0 ± 80.1, *p* = 0.664; IL8: 149.4 ± 154.2 vs. 121.6 ± 101.6, *p* = 0.176; and IL10: 12.9 ± 65.2 vs. 6.0 ± 2.4, *p* = 0.387, respectively). At the postoperative day 7, no significant difference was found in the serum IL1, IL2, IL6, IL8, IL10, and TNF-*α* concentrations between groups (Figures 1(a)–1(f); *p* > 0.05 for all).

**Figure 2:**
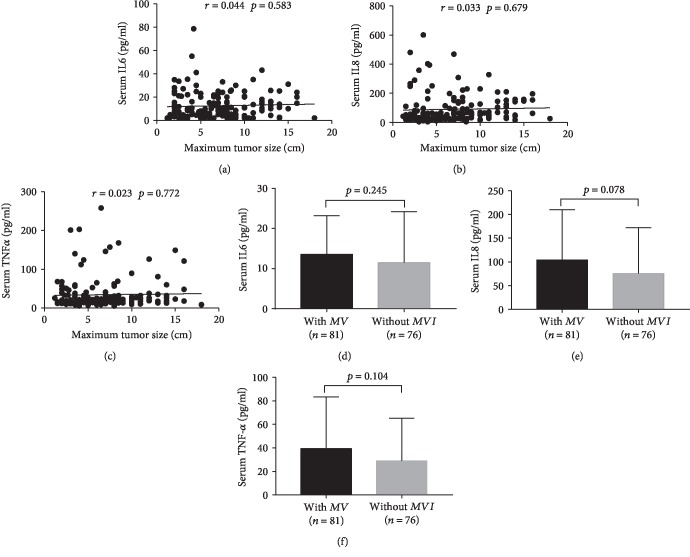
The correlations between maximum tumor size, MVI, and preoperative serum IL6, IL8, and TNF-*α* levels. Preoperative serum IL6 (a), IL8 (b), and TNF-*α* (c) are not associated with maximum tumor size. There is no difference between preoperative serum IL6 (d), IL8 (e), and TNF-*α* (f) levels in patients with MVI and patients without MVI.

As shown in Table 1, the maximum tumor size of the recurrence group was distinctly bigger than the nonrecurrence group, and MVI is more common in the recurrence group. In order to exclude the impacts that maximum tumor size and MVI imposed on preoperative serum inflammatory mediator levels, we examined the correlations between them by Pearson *r*. Pearson *r* analysis revealed that preoperative serum IL6, IL8, and TNF-*α* levels were not associated with maximum tumor size (Figures 2(a)–2(c), IL6: *r* = 0.051, *p* = 0.529; IL8: *r* = 0.118, *p* = 0.141; and TNF-*α*: *r* = 0.029, *p* = 0.722, respectively); furthermore, there was no relationship between preoperative serum IL6, IL8, and TNF-*α* levels and MVI status (Figures 2(d)–2(f), *p* = 0.245, *p* = 0.078, and *p* = 0.104, respectively).

### 3.3. Survival and Risk Factor

**Figure 3:**
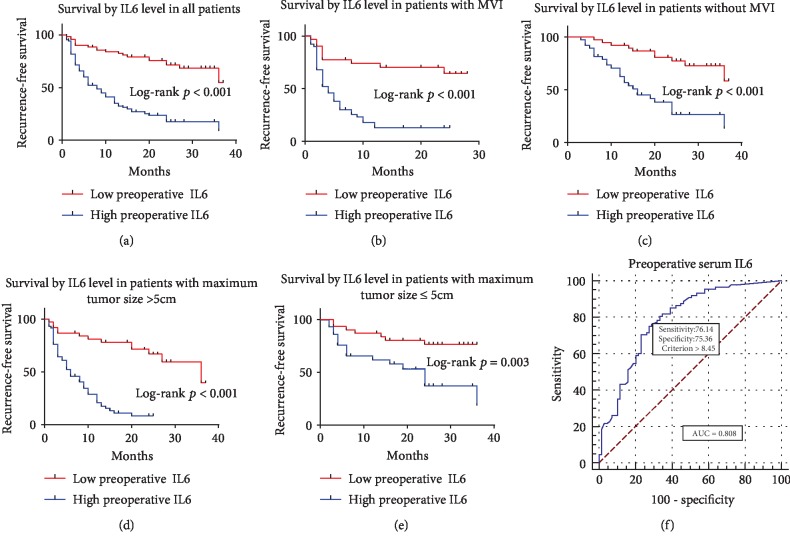
Kaplan-Meier curves for RFS of patients who had low preoperative serum IL6 level versus high preoperative serum IL6 level. (a) Kaplan-Meier curves for RFS in all patients by preoperative serum IL6 level. (b, c) Kaplan-Meier curves for RFS in patients with MVI or without MVI by preoperative serum IL6 level. (d, e) Kaplan-Meier curves for RFS in patients with maximum tumor size > 5 cm or ≤5 cm by preoperative serum IL6 level. (f) ROC curve for preoperative serum IL6 level.

**Figure 4:**
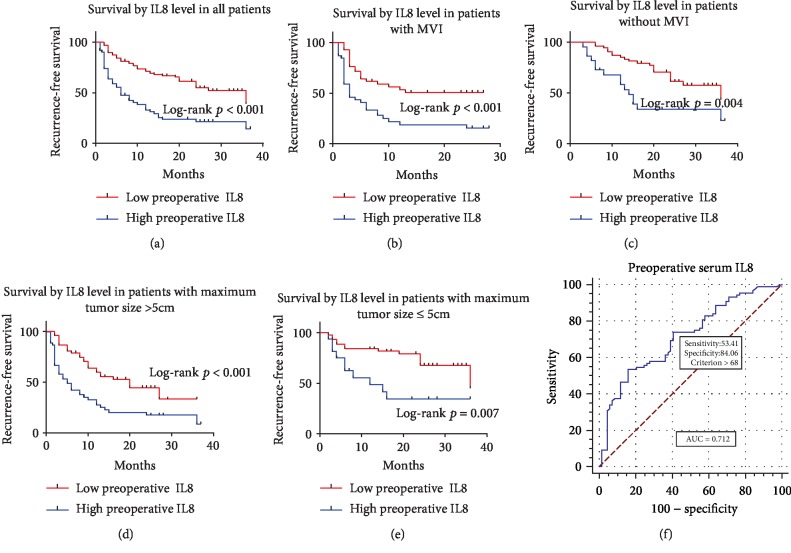
Kaplan-Meier curves for RFS of patients who had low preoperative serum IL8 level versus high preoperative serum IL8 level. (a) Kaplan-Meier curves for RFS in all patients by preoperative serum IL8 level. (b, c) Kaplan-Meier curves for RFS in patients with MVI or without MVI by preoperative serum IL8 level. (d, e) Kaplan-Meier curves for RFS in patients with maximum tumor size > 5 cm or ≤ 5 cm by preoperative serum IL8 level. (f) ROC curve for preoperative serum IL8 level.

**Figure 5:**
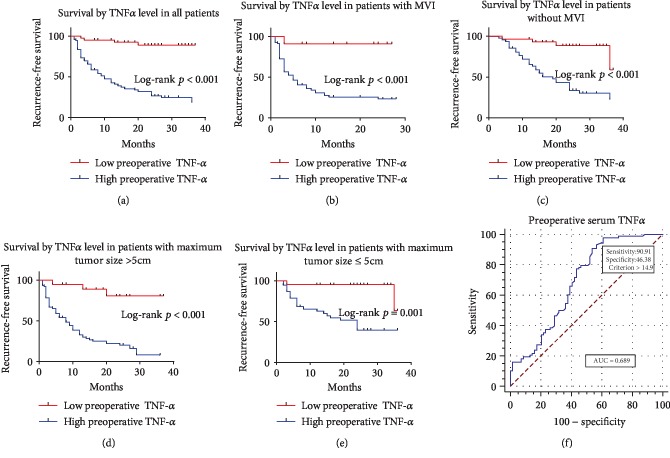
Kaplan-Meier curves for RFS of patients who had low preoperative serum TNF-*α* level versus high preoperative serum TNF-*α* level. (a) Kaplan-Meier curves for RFS in all patients by preoperative serum TNF-*α* level. (b, c) Kaplan-Meier curves for RFS in patients with MVI or without MVI by preoperative serum TNF-*α* level. (d, e) Kaplan-Meier curves for RFS in patients with maximum tumor size > 5 cm or ≤5 cm by preoperative serum TNF-*α* level. (f) ROC curve for preoperative serum TNF-*α* level.

Figures 3–5 illustrated corresponding recurrence-free survival rates on the basis of preoperative serum IL6, IL8, and TNF-*α* levels. ROC curve analysis (AUC = 0.808, 0.712, and 0.689, respectively) found that the optimal preoperative serum IL6, IL8, and TNF-*α* level cut-off values for the presence of recurrence were 8.45, 68, and 14.9, respectively (Figures 3(f), 4(f), and 5(f)). Consequently, patients were divided into groups with low (≤8.45 pg/ml; *n* = 73) or high (>8.45 pg/ml; *n* = 84) preoperative IL6, groups with low (≤68 pg/ml; *n* = 99) or high (>68 pg/ml; *n* = 58) preoperative IL8, and groups with low (≤14.9 pg/ml; *n* = 40) or high (>14.9 pg/ml; *n* = 117) preoperative TNF-*α*. High preoperative serum IL6, IL8, and TNF-*α* levels were distinctly correlated with shorter RFS (*p* < 0.001 for all) after radical hepatectomy (Figures 3(a), 4(a), and 5(a)). Furthermore, whether in patients with MVI or without MVI, high preoperative serum IL6, IL8, and TNF-*α* levels were distinctly correlated with shorter RFS (Figures 3(b), 3(c), 4(b), 4(c), 5(b), and 5(c)). Finally, whether in patients with maximum tumor size ≤ 5 cm or >5 cm, high preoperative serum IL6, IL8, and TNF-*α* levels were distinctly correlated with shorter RFS (Figures 3(d), 3(e), 4(d), 4(e), 5(d), and 5(e)).

In order to confirm the risk factors of RFS in HCC patients, Cox proportional hazard analysis was performed with clinical factors (age, gender, maximum tumor size, MVI, cirrhosis, differentiation, HBsAg status, and AFP) and preoperative serum IL6, IL8, and TNF-*α* levels. Univariate analysis revealed that preoperative serum IL6 > 8.45 pg/ml, preoperative serum IL8 > 68 pg/ml, preoperative serum TNF − *α* > 14.9 pg/ml, MVI, and maximum tumor size > 5 cm were significantly correlated with RFS in HCC patients who accepted radical hepatectomy (Table 2). Multivariate analysis revealed that preoperative serum IL6 > 8.45 pg/ml (HR 4.42, 95% CI 2.58-7.58, *p* < 0.001), preoperative serum IL8 > 68 pg/ml (HR 1.73, 95% CI 1.12-2.68, *p* = 0.013), preoperative serum TNF − *α* > 14.9 pg/ml (HR 5.48, 95% CI 2.18-13.81, *p* = 0.004), MVI (HR 1.86, 95% CI 1.15-2.99, *p* = 0.011), and maximum tumor size > 6 cm (HR 1.72, 95% CI 1.03-2.89, *p* = 0.039) were independent risk factors of recurrence-free survival in HCC patients (Table 2).

## 4. Discussion

Our results showed that preoperative serum IL6, IL8, and TNF-*α* levels were significantly correlated with the postoperative tumor recurrence risk of the HCC patients, and both univariate and multivariate analyses showed that preoperative serum IL6 > 8.45 pg/ml, preoperative serum IL8 > 68 pg/ml, preoperative serum TNF − *α* > 14.9 pg/ml, MVI, and maximum tumor size > 6 cm were independent predictors of recurrence-free survival. Furthermore, whether in patients with MVI or without MVI, or in patients with maximum tumor size > 5 cm or ≤5 cm, high preoperative serum IL6, IL8, and TNF-*α* levels were distinctly correlated with shorter RFS. Therefore, we concluded that the preoperative serum IL-6, IL8, and TNF-*α* levels may serve as potential predictors for the clinical outcome of HCC patients.

It is well known that inflammation and immune mediators play an important role in cancer progression [2, 3]. Casadei Gardini et al. showed that the systemic immune-inflammation index (SII, calculated as platelet count∗neutrophil count/lymphocyte count) was negatively associated with the progression-free survival and overall survival in advanced HCC patients receiving sorafenib [22]. As an essential element of inflammatory response, IL-6 is one of the best characterized protumorigenic cytokines; it was not only involved in immune regulation but also associated with tumor immune microenvironment. Within the body, many kinds of cells, such as T cells, endothelial cells, macrophages, and tumor cells, can synthesize IL6 [23]. In previous studies, Bromberg and Wang [23] and He and Karin [24] showed that IL-6 could contribute to the HCC development by activating the nuclear factor-*κ*B, the signal transducer and activator of transcription. Furthermore, Porta et al. [25] showed that circulating IL6 could be regarded as a tumor marker for HCC, and Wong et al. [26] concluded that high serum IL6 level could predict future HCC development in patients with chronic hepatitis B. In line with these studies, the present study also revealed that a high preoperative serum IL-6 level is correlated with tumor recurrence risk of patients with HCC. Altogether, the preoperative serum IL-6 level may serve as a predictor for the tumor recurrence risk of HCC patients.

In addition to IL6, we also found that high preoperative serum IL8 and TNF-*α* levels were significantly correlated with RFS. The expression of IL-8 was increased in cancer cells and tumor-associated macrophages, suggesting that IL-8 may play a crucial role in the tumor microenvironment. Clinically, some studies showed that IL8 was involved in the evolvement of cancer. For example, Welling et al. [27] concluded that a high preoperative serum IL-8 is correlated with the diagnosis of HCC and is an independent predictor of survival. Moreover, Lee et al. [28] showed that IL-8 and its receptor CXCR2 can contribute to the evolvement of colon cancer and promote the metastasis. Mechanistically, a previous study suggested that IL8 could activate the expression of FOXC1, leading to activation of CXCR1 and CCL2, which can promote inflammation and enhance the malignance of HCC cells [29]. There are also many studies that showed the strong relationship between TNF-*α* and the development of liver cancer [19]. Altogether, preoperative serum IL-8 and TNF-*α* levels may serve as predictors for the tumor recurrence risk of HCC patients.

Preoperative serum inflammatory mediators (such as IL6, IL8, and TNF-*α*) may be associated with liver fibrosis and cirrhosis. As shown in previous studies, Xiang et al. [30] suggested that the HLF/IL-6/STAT3 pathway plays an important role in liver fibrosis, and Dirchwolf et al. [31] revealed that the serum inflammatory mediator (such as IL-6, IL-7, IL-8, IL-10, IL 12, and TNF-*α*) levels in cirrhosis patients were significantly higher than those in healthy controls. Furthermore, D'Anzeo et al. demonstrated that various aberrantly expressed miRNAs (such as miR122, miR-494, and miR-429) are correlated with the prognosis of HCC patients. Therefore, further study is needed to explore the signaling pathway that connects inflammatory mediators with miRNAs, and targeting those pathways may contribute to the treatment of HCC [32].

Though previous studies had investigated the association between inflammatory mediators (such as IL1, IL4, IL6, IL8, and IL10) and the prognosis of patients with HCC, few studies examined these cytokines at the same time. Differently, the present study strictly collected data of perioperative inflammatory mediators, including IL1, IL2, IL6, IL8, IL10, and TNF-*α*, and observed the dynamic changes of these cytokines between groups. Furthermore, our findings combined with other studies' results show that preoperative serum IL6, IL8, and TNF-*α* levels play an important role in the development and progression of HCC. Therefore, these cytokines not only can predict the prognosis of HCC patient but also can be regarded as therapeutic targets.

Our study has several limitations. First, it is a retrospective study, so we cannot avoid some potential bias. Second, this study only included patients from one centre, and its sample is relatively small, so IL6, IL-8, and TNF-*α* should not be used as new predictors until large prospective studies can be performed. Last, a previous study showed that IL6 was not related to the clinical outcome in patients with resectable hepatocellular carcinoma [33], so our results need to be further validated in well-designed future studies.

## 5. Conclusion

Our findings show that high preoperative serum IL6, IL8, and TNF-*α* levels were significantly correlated with the postoperative tumor recurrence risk of the HCC patients. Therefore, we concluded that preoperative sera IL6, IL8, and TNF-*α* may predict the postoperative recurrence of hepatocellular carcinoma.

## Figures and Tables

**Table 1:** Demographics and baseline characteristics of the patients from the recurrence group and the nonrecurrence group.

Factor	Recurrence group (*n* = 88)	Nonrecurrence group (*n* = 69)	*p* value
Age (year)	51.6 ± 9.9	53.9 ± 9.5	0.124
Male/female	74/14	55/14	0.477
Tumor size (cm)	8.1 ± 3.9	5.5 ± 3.2	<0.001
MVI (present/absent)	52/36	29/40	0.034
Child-Pugh, A/B	84/4	65/4	0.732
Differentiation (poor/well)	32/56	17/52	0.116
Cirrhosis, yes/no	65/23	51/18	0.994
HBsAg, yes/no	58/30	46/23	0.921
AFP	113.5 (13.9-1434.3)	150.0 (21.9-627.2)	0.857
BMI	24.4 ± 3.5	24.4 ± 2.9	0.984
ALT	48.3 (24.7-87.1)	32.4 (22.2-627.2)	0.157
AST	44.1 (24.0-102.7)	31.2 (20.1-59.9)	0.052
TBIL	14.7 (10.3-21.8)	14.2 (10.8-17.4)	0.278
Operative procedure			0.992
Extended hemihepatotectomy	5	5	
Hemihepatotectomy	11	9	
Sectionectomy	25	20	
Segmentectomy	9	6	
Laparoscopic approach	38	29	
Pringle maneuver	50/38	42/27	0.609
Operative time (min)	240.0 (200.0-293.8)	255.0 (200.0-285.0)	0.479
Total blood loss (ml)	500.0 (212.5-700.0)	400.0 (200.0-600.0)	0.226

Note: data are presented as the median (IQR) or mean ± SD. Abbreviations: BMI: body mass index; ALT: alanine aminotransferase; AST: aspartate aminotransferase; AFP: *α*-fetoprotein; TBIL: total bilirubin; HBsAg: hepatitis B virus surface antigen; MVI: microvascular invasion.

**Table 2:** Univariate and multivariate analyses of risk factors for recurrence-free survival.

Factor	Univariable analysis		Multivariable analysis	
	HR (95% CI)	*p* value	HR (95% CI)	*p* value
Age (years)				
≥60	0.99 (0.61-1.60)	0.964		
<60				
Gender				
Male	0.78 (0.44-1.38)	0.398		
Female				
HBV				
Yes	0.97 (0.64-1.49)	0.902		
No				
Cirrhosis				
Yes	0.91 (0.56-1.46)	0.681		
No				
Differentiation				
Poor	0.68 (0.44-1.04)	0.078		
Well				
AFP (*μ*g/l)				
>636	1.51 (0.97-2.34)	0.069		
≤636				
Maximum tumor size (cm)				
≥5	2.37 (1.48-3.81)	<0.001	1.72 (1.03-2.89)	0.039
<5				
MVI				
Yes	2.47 (1.59-3.83)	<0.001	1.86 (1.15-2.99)	0.011
No				
Preoperative serum IL6 (pg/ml)				
>8.45	4.69 (2.82-7.81)	<0.001	4.42 (2.58-7.58)	<0.001
≤8.45				
Preoperative serum IL8 (pg/ml)				
>68	2.80 (1.84-4.28)	<0.001	1.73 (1.12-2.68)	0.013
≤68				
Preoperative serum TNF*α* (pg/ml)				
>14.9	9.15 (3.70-22.60)	<0.001	5.48 (2.18-13.81)	<0.001
≤14.9				

Abbreviations: AFP: *α*-fetoprotein; HR: hazard rate; CI: confidence interval; MVI: microvascular invasion.

## Data Availability

The data used to support the findings of this study are available from the corresponding author upon request.
